# Different Substrate Preferences Help Closely Related Bacteria To Coexist in the Gut

**DOI:** 10.1128/mBio.01824-17

**Published:** 2017-11-07

**Authors:** Petra Louis

**Affiliations:** The Rowett Institute, University of Aberdeen, Aberdeen, United Kingdom

**Keywords:** *Bacteroides*, community diversity, competition, glycan, microbiota

## Abstract

Many factors shape the ability of different microbes to coexist in microbial communities. In the human gut, dietary and host-derived nutrients largely drive microbial community structure. How gut microbes with very similar nutrient profiles are able to coexist over time within the same host is not fully understood. Tuncil et al. (mBio 8:e01068-17, 2017, https://doi.org/10.1128/mBio.01068-17) explored glycan prioritization in two closely related human gut bacteria, *Bacteroides ovatus* and *Bacteroides thetaiotaomicron*, on complex glycan mixtures that both organisms can degrade. Determining depletion of the individual glycans over time in pure cultures and cocultures revealed that the bacteria seem to have hardwired differences in their preferences for different glycans which likely contribute to their stable coexistence. The researchers also established that gene expression changes of the corresponding polysaccharide utilization loci did not always mirror glycan depletion, which highlights that additional regulatory mechanisms must be present.

## COMMENTARY

The human large intestinal microbiota largely thrives on dietary carbohydrates originating from plant and fungal sources that are indigestible in the upper gut. Those glycans consist of a diverse range of structurally different polymers and oligomers that differ in their monosaccharide constituents, types of glycosidic linkage, occurrence and degree of branching, chain lengths, and presence of other bound constituents such as phenolic residues. This assorted glycan mixture, together with some host-derived molecules, sustains a highly diverse microbial community that consists of hundreds of different species (1). Microbial diversity is likely maintained over time by different species occupying different ecological niches, both with regard to which glycans they can utilize and with regard to their response to other environmental factors such as pH or oxygen exposure. However, this is not sufficient to explain the level of diversity observed, as closely related bacteria with very similar properties are frequently found to coexist in the same individual. This is exemplified by bacteria of the genus *Bacteroides*, which tend to be generalists that thrive on many different soluble glycans and frequently coexist within individuals.

The presence of a highly diverse microbiota composition has been linked to health, as diversity is often found to be reduced in disease states such as inflammatory bowel disease (IBD) and metabolic syndrome (2). Thus, a better understanding of how diversity is maintained is crucial for the development of efficient strategies to preserve or regenerate a diverse microbiota, in particular, by strategic delivery of dietary glycans. A more radical approach to modulate the microbiota is to essentially replace it with a more beneficial microbial community. That this can be a very effective means of improving health has been demonstrated by fecal transplantation trials performed with individuals suffering from recurring *Clostridium difficile* infections. Transplantation of feces from a healthy donor has been shown to lead to remission in over 90% of patients in several studies to date (3). However, it would be preferable to replace the complex donor fecal microbiota with defined mixtures of known and well-characterized gut bacteria to minimize the risk of transferring unknown infectious agents and to explore the development of more targeted approaches for treatment of other gut diseases such as IBD. To maximize the chance of success of such treatments, it is crucial to have a sound understanding of which species are most likely to cocolonize and thrive together over extended time periods.

Tuncil et al. addressed the question of how microbes with similar glycan utilization profiles coexist by examining glycan degradation in two closely related gut species, *Bacteroides ovatus* and *Bacteroides thetaiotaomicron* (4). *Bacteroidetes* species contain multiple polysaccharide utilization loci (PULs), gene clusters comprising glycan binding proteins, sensors, transporters, enzymes for glycan degradation, and transcription factors that are coregulated in response to their specific glycan (5). The researchers combined measurements of glycan depletion in cultures of *B. ovatus* and *B. thetaiotaomicron* grown on glycan mixtures with gene expression analysis of the corresponding PULs. Six glycans were chosen that can be utilized by both species and can selectively be detected based on their carbohydrate structure (monosaccharide composition and/or linkages). This revealed that gene expression alone is not sufficient to predict glycan depletion, as, for certain glycans, their depletion was delayed relative to an increase in expression of their corresponding PUL. Thus, other mechanisms must contribute to effective glycan degradation in addition to gene expression.

The order and speed with which different glycans were depleted differed between the two strains. Furthermore, gene expression patterns for each bacterium were very similar in cocultures of both strains compared to their respective monocultures, indicating that the bacteria are “programmed to utilize glycans in a hierarchical order” regardless of whether competing bacteria are present or not. Both strains persisted well with roughly even levels of abundance over the first 10 h of the coculture, but then *B. ovatus* outcompeted *B. thetaiotaomicron*. In cocultures containing each glycan on its own, on the other hand, *B. ovatus* always outcompeted *B. thetaiotaomicron*, and this process for most glycans started much earlier than in the cultures containing all six glycans. This indicates that the extended period of coexistence between the two strains in cultures containing the glycan mixture is due to their different hierarchical orders by which they consume the glycans present, which avoids direct substrate competition. Thus, the hardwiring of preferences for individual glycans by different bacteria seems to facilitate their coexistence in microbial communities.

The ability of *B. ovatus* to outcompete *B. thetaiotaomicron* on arabinan was particularly intriguing, as *B. ovatus* showed poor growth of this glycan in monoculture. The researchers therefore tested whether *B. thetaiotaomicron* provides glycan breakdown products to *B. ovatus*. *B. thetaiotaomicron* released arabinan oligosaccharides into the medium during growth on arabinan. Furthermore, *B. ovatus* was able to grow well on arabinose and arabinobiose and also grew on exponential-phase media from *B. thetaiotaomicron*, reaching higher growth rates than *B. thetaiotaomicron* with media from the late exponential phase. The authors also reported that this effect was not due to the provision of capsular polysaccharides. Thus, *B. thetaiotaomicron* appears to facilitate growth of *B. ovatus* in the coculture on arabinan by cross-feeding glycan breakdown products.

The glycans chosen here belong to structurally different polysaccharides (amylopectin, chondroitin sulfate, polygalacturonic acid, rhamnogalacturonan I, pectic galactan, and arabinan), and the issue arises whether corresponding derivatives with a simpler molecular structure would be prioritized differently in glycan mixtures. The researchers addressed this issue by focusing on alpha-glucan breakdown of *B. ovatus*, as they established that this strain grew much better on malto-oligosaccharides than on a range of different starches. Amylopectin had ranked fifth in the order in which the different glycans were degraded in the glycan mixture, but when it was replaced by maltohexaose, the alpha-glycan rose to the fourth rank and displaced rhamnogalacturonan I from the fourth to fifth rank, whereas the order of the glycans ranked higher was not affected. This change was mirrored in the gene expression of the corresponding PULs. Thus, despite maltohexaose being an excellent growth substrate in pure culture, it did not replace highly ranked structurally different glycans when presented as part of a mixture. The degree of branching in different starches may also affect glycan utilization, as it was established that the linear amylose fraction was utilized preferentially over branched amylopectin by *B. ovatus*.

The results of the study reported by Tuncil et al. suggest that hardwired differences in hierarchies of glycan preferences enable closely related bacteria with similar glycan degradation capacities to coexist with each other, which contributes to the maintenance of highly diverse microbial communities. It would be interesting to extend this work to other *Bacteroides* species and combine it with co-occurrence network analysis of the microbiota of different individuals to establish whether the magnitude of these programmed differences in glycan preferences between species translates to their frequency of coexistence.

A particular strength of the study by Tuncil et al. (4) is that gene expression analysis was complemented by measurements of the actual disappearance of the respective glycans, which revealed that gene expression of the cognate PUL alone is not sufficient for effective breakdown of some glycans. This may be due to a range of different posttranscriptional effects precluding effective translation or processing of the corresponding proteins. Thus, *cis*-encoded small RNAs that may interfere with efficient protein expression at the level of transcript stability or translation were recently described in *Bacteroides fragilis* (6). Global analysis of the transcriptome and/or proteome is required to enable better understanding of the underlying processes leading to the delay between gene expression and glycan degradation. A more global analysis may also facilitate a deeper understanding of why maltohexaose is prioritized only marginally more highly by *B. ovatus* than the more complex amylopectin, despite the fact that it is a much better substrate for this organism, as such analyses could reveal insights into other gene products that may be required and into global regulatory processes underlying glycan prioritization.

*Bacteroides* species tend to be generalists that can grow on a wide variety of glycans, but other bacteria in the gut follow different nutritional strategies. For example, *Roseburia* species within the *Firmicutes* have recently been shown to contain gene arrangements similar to those seen with PULs (termed Gram-positive [gp] PULs); however, different *Roseburia* species show only limited overlap with respect to the glycans that they can grow on and appear to specialize on different glycans (7). Some other *Firmicutes* seem to have taken this a step further, exemplified by *Ruminococcus bromii*, which specializes in resistant starch breakdown and constitutively expresses the corresponding genes even in the absence of starch (8). Cross-feeding of glycan breakdown products or other metabolites, with the example of arabinan oligosaccharide cross-feeding from *B. thetaiotaomicron* to *B. ovatus* as shown by Tuncil et al. (4), also likely plays an important role in maintaining diversity. Furthermore, other interactions between bacteria, including the production of antimicrobial compounds and quorum sensing networks, must also be considered in studying microbial coexistence. It also has to be kept in mind that nutrient input constantly changes in qualitative and quantitative terms in humans, which likely leads to fluctuations in the abundances of specific species but may rarely lead to a complete washout of species from the system if a diet diverse in different nondigestible glycans is consumed. Bacteriophages, which are known to be highly abundant in the gut but remain understudied to date, are expected to contribute a further layer of fluctuation in the abundances of different species which modifies the overall dynamic between species (9). Last but not least, spatial separation between competing species is also increasingly being recognized as an important factor in maintaining diversity in the community (10).

## References

[1] Flint HJ, Scott KP, Duncan SH, Louis P, Forano E 2012 Microbial degradation of complex carbohydrates in the gut. Gut Microbes 3:289–306. doi:10.4161/gmic.19897. 22572875 PMC3463488

[2] Karkman A, Lehtimäki J, Ruokolainen L 2017 The ecology of human microbiota: dynamics and diversity in health and disease. Ann N Y Acad Sci 1399:78–92. doi:10.1111/nyas.13326. 28319653

[3] Quraishi MN, Widlak M, Bhala N, Moore D, Price M, Sharma N, Iqbal TH 2017 Systematic review with meta-analysis: the efficacy of faecal microbiota transplantation for the treatment of recurrent and refractory *Clostridium difficile* infection. Aliment Pharmacol Ther 46:479–493. doi:10.1111/apt.14201. 28707337

[4] Tuncil YE, Xiao Y, Porter NT, Reuhs BL, Martens EC, Hamaker BR 2017 Reciprocal prioritization to dietary glycans by gut bacteria acts in a competitive environment, promoting stable coexistence. mBio 8:e01068-17. doi:10.1128/mBio.01068-17. 29018117 PMC5635687

[5] Grondin JM, Tamura K, Déjean G, Abbott DW, Brumer H 2017 Polysaccharide utilization loci: fueling microbial communities. J Bacteriol 199:e0086016. doi:10.1128/JB.00860-16. PMC5512228 28138099

[6] Cao Y, Förstner KU, Vogel J, Smith CJ 2016 *cis*-encoded small RNAs, a conserved mechanism for repression of polysaccharids utilization in *Bacteroides*. J Bacteriol 198:2410–2418. doi:10.1128/JB.00381-16. 27353652 PMC4999932

[7] Sheridan PO, Martin JC, Lawley TD, Browne HP, Harris HMB, Bernalier-Donadille A, Duncan SH, O’Toole PW, Scott KP, Flint HJ 2016 Polysaccharide utilization loci and nutritional specialization in a dominant group of butyrate-producing human colonic Firmicutes. Microb Genom 2:e000043. doi:10.1099/mgen.0.000043. 28348841 PMC5320581

[8] Ze X, Ben David Y, Laverde-Gomez JA, Dassa B, Sheridan PO, Duncan SH, Louis P, Henrissat B, Juge N, Koropatkin NM, Bayer EA, Flint HJ 2015 Unique organization of extracellular amylases into amylosomes in the resistant starch-utilizing human colonic *Firmicutes* bacterium *Ruminococcus bromii*. mBio 6:e01425-15. doi:10.1128/mBio.01058-15. 26419877 PMC4611034

[9] Mirzaei MK, Maurice CF 2017 Ménage à trois in the human gut: interactions between host, bacteria and phages. Nat Rev Microbiol 15:397–408. doi:10.1038/nrmicro.2017.30. 28461690

[10] Pereira FC, Berry D 2017 Microbial nutrient niches in the gut. Environ Microbiol 19:1366–1378. doi:10.1111/1462-2920.13659. 28035742 PMC5412925

